# Quantum Mechanical Analysis Based on Perturbation Theory of CdSe/ZnS Quantum-Dot Light-Emission Properties

**DOI:** 10.3390/nano12203590

**Published:** 2022-10-13

**Authors:** Honyeon Lee, Dongjin Kim

**Affiliations:** Department of Electronic Materials, Devices and Equipment Engineering, Soonchunhyang University, Asan 31538, Korea

**Keywords:** quantum dots, CdSe/ZnS, core diameter, electric field intensity, energy levels, perturbation, quantum mechanics, numerical analysis

## Abstract

A simulation of quantum dot (QD) energy levels was designed to reproduce a quantum mechanical analytic method based on perturbation theory. A Schrödinger equation describing an electron–hole pair in a QD was solved, in consideration of the heterogeneity of the material parameters of the core and shell. The equation was solved numerically using single-particle basis sets to obtain the eigenstates and energies. This approach reproduced an analytic solution based on perturbation theory, while the calculation was performed using a numerical method. Owing to the effectiveness of the method, QD behavior according to the core diameter and external electric field intensity could be investigated reliably and easily. A 9.2 nm diameter CdSe/ZnS QD with a 4.2 nm diameter core and 2.5 nm thick shell emitted a 530 nm green light, according to an analysis of the effects of core diameter on energy levels. A 4 nm redshift at $5.4\times 10^{5}$ V/cm electric field intensity was found while investigating the effects of external electric field on energy levels. These values agree well with previously reported experimental results. In addition to the energy levels and light emission wavelengths, the spatial distributions of wavefunctions were obtained. This analysis method is widely applicable for studying QD characteristics with varying structure and material compositions and should aid the development of high-performance QD technologies.

## 1. Introduction

A quantum dot (QD) is an excellent optoelectronic material among various nanoparticles [1,2,3,4]. Its light emission spectra are sharp and easily controllable by varying its diameter [5]. In addition, stable QD light-emitting layers (EMLs) can be fabricated because of the inorganic nature of QDs. Given these advantages, there have been continuous efforts to develop high-performance QDs and light-emitting diodes using QD EMLs, with the aim of replacing organic light-emitting diodes (OLEDs) with quantum-dot light-emitting diodes (QLEDs) [6,7,8,9,10]. However, research on QDs and QLEDs is in the early stage; thus, their lifetime [11,12] and efficiency [13,14] should be improved further before commercial application of QLEDs. A detailed analysis of the energy levels of QDs is required, as these determine the optoelectronic characteristics. Quantum confinement effects modulate QD energy levels and luminescence properties [15,16]. In addition, the quantum-confined Stark effect (QCSE) [17,18] arising from the voltage bias influences the electroluminescence properties of QDs. Despite their importance, it is difficult to analyze quantum effects experimentally. Hence, theoretical and/or numerical analyses are required [19,20]. Quantum phenomena involving QDs can be described by the Schrödinger equation, which is solved in consideration of the potential profile, material properties (such as effective masses and dielectric constants), and the Coulomb interactions in QDs. The entire Hamiltonian can be solved using the finite difference method (FDM) or finite element method (FEM); these methods are firmly established in terms of their ability to provide sophisticated numerical results [21,22]. However, analytic solutions are more suitable for interpreting quantum mechanical behavior. A perturbation method is used as a practical way to obtain analytic solutions. The unperturbed Hamiltonian is composed of single-particle Hamiltonian terms representing an electron and hole. Profiles of the electric potential, effective mass, and dielectric constant, and the electron–hole Coulomb interaction, are considered in the perturbation terms. Despite the usefulness of analytic solutions based on this perturbation method, extensive effort is required to attain analytical solutions under varying conditions, such as QD diameter, electric field intensity, and material properties. A semi-analytic method may overcome this, as it imitates the analytic method using a numerical method [23]. In the previous work, the Schrödinger equation for a QD was solved based on single-particle Schrödinger equations, using perturbation terms corresponding the electron–hole Coulomb energy and potential energy related with energy-band profiles. However, profiles of the effective mass and dielectric constant were ignored to simplify the analysis, which may have caused unnecessary errors. In this work, the semi-analytic method is improved significantly for more accurate analysis. The profiles of effective mass and dielectric constant are included in the perturbation terms, and image charges are considered when calculating the Coulomb energy. Using this approach, an efficient method for understanding quantum phenomena in QDs is derived. The dependency of the light wavelength emitted from QDs and wavefunction shapes in QDs on the QD diameter and external electric field intensity was examined using the proposed approach. To verify the effectiveness of the proposed method, the properties of CdSe/ZnS core/shell QDs were analyzed using this method. The CdSe/ZnS has become a major QD structure because of its easy synthesis and high performance [24,25,26]. Therefore, reliable comparative experimental data of CdSe/ZnS QDs can be obtained relatively easily. This makes CdSe/ZnS QDs a suitable test vehicle for this work.

## 2. Methods

The Schrödinger equation describing the electron–hole system in a QD was solved and the light-emission properties were examined. The Schrödinger equation is composed of kinetic energy and potential energy terms. The potential energy term has two elements: one is associated with the energy band profile, and the other with the Coulomb interaction. As a QD has a core and shell made of different materials, different values for the effective mass and dielectric constant of each layer are considered.

The system was approximated by one-dimensional equations for efficient calculation and analysis. Equations (1)–(4) define Hamiltonian terms.

(1) $$\hat{H}={\hat{T}}_{e}+{\hat{T}}_{h}+{\hat{V}}_{e}+{\hat{V}}_{h}+{\hat{H}}_{int},$$

(2) $${\hat{T}}_{e}\left(x_{e}\right)=-\frac{\hbar^{2}}{2m_{e}^{*}}\frac{\partial^{2}}{\partial x_{e}^{2}},$$

(3) $${\hat{T}}_{h}\left(x_{h}\right)=-\frac{\hbar^{2}}{2m_{h}^{*}}\frac{\partial^{2}}{\partial x_{h}^{2}},$$

(4) $${\hat{H}}_{int}=-\frac{1}{3}\frac{1}{4\pi \epsilon }\frac{e^{2}}{\sqrt{3}\left|x_{e}-x_{h}\right|},$$

where $\hat{H}$, ${\hat{T}}_{e}$, ${\hat{T}}_{h}$, ${\hat{V}}_{e}$, ${\hat{V}}_{h}$, and ${\hat{H}}_{int}$ represent the total Hamiltonian, electron kinetic energy, hole kinetic energy, electron potential energy, hole potential energy, and Coulomb interaction energy, respectively. $m_{e}^{*}$, $m_{h}^{*}$, ϵ, $x_{e}$, and $x_{h}$ represent the electron effective mass, hole effective mass, dielectric constant, electron position, and hole position, respectively.

The values of $m_{e}^{*}$, $m_{h}^{*}$, and ϵ change according to the regions of QD core and shell. For the symmetric case, the three-dimensional electron–hole distance $\left|\vec{r_{e}}-\vec{r_{h}}\right|$ is $\sqrt{3}\left|x_{e}-x_{h}\right|$, where $\left|x_{e}-x_{h}\right|$ is the one-dimensional electron–hole distance. $\vec{r_{e}}$ and $\vec{r_{h}}$ are the position vectors of electron and hole in three dimensions. Hence, the three-dimensional Coulomb energy can be approximated as $-\frac{1}{4\pi \epsilon }\frac{e^{2}}{\sqrt{3}\left|x_{e}-x_{h}\right|}$. The one-dimensional Coulomb energy is one-third of the three-dimensional Coulomb energy for the symmetric case. As a result, Equation (4) is obtained.

Two single-particle basis sets were combined to produce the QD electron–hole system basis set; one single-particle basis set was the electron basis set and the other was the hole basis set. The number of single-particle bases was restricted to a finite number of *n_max_*. The single particle kinetic energy operator for an infinite well was used to define the momentum basis set; the well width was the same as the total width of the QD. The QD core effective mass values were used for this momentum basis set. The QD shell effective mass values were reflected in the perturbation term. The tensor product of the electron and hole basis sets defines the system basis as:

(5) $$\left|n_{e},n_{h}\rangle \right.=\left|n_{e}\rangle \right.⨂\left|n_{h}\rangle \right.\;for\;n_{e},n_{h}\in \left\{1,2,3,\cdots ,n_{max}\right\},$$

where $\left|n_{e}\rangle \right.$ and $\left|n_{h}\rangle \right.$ are the electron and hole bases, respectively. A position basis set was also defined for describing the potential energy terms. When defining the position basis, the potential well was equally divided by *n_max_* points. The position basis $\left|j\right.\rangle$ is expressed as $\sqrt{\Delta }\sum_{n=1}^{n_{max}}\langle n|x_{j}\rangle \left|n,\rangle \right.$, where $\left|n\right.\rangle$, $\left|x_{j}\rangle \right.$, and Δ are the single-particle momentum basis, Dirac position basis at position $x_{j}$, and spacing between *n_max_* points, respectively [27]. The system basis set is defined as:

(6) $$\left|j_{e},j_{h}\rangle \right.=\left|j_{e}\rangle \right.⨂\left|j_{h}\rangle \right.\;\mathrm{for}\;j_{e},j_{h}\in \left\{1,2,3,\cdots ,n_{max}\right\},$$

where $\left|j_{e}\rangle \right.$ and $\left|j_{h}\rangle \right.$ are the position bases of electron and hole, respectively.

The unperturbed kinetic energy of an electron, ${\hat{T}}_{e0}=-\frac{\hbar^{2}}{2m_{ec}^{*}}\frac{\partial^{2}}{\partial x_{e}^{2}}$, and of a hole, ${\hat{T}}_{h0}=-\frac{\hbar^{2}}{2m_{hc}^{*}}\frac{\partial^{2}}{\partial x_{h}^{2}}$, are represented by the momentum basis set as $\sum_{n_{e}=1}^{n_{max}}\left.\langle n_{e}\right|{\hat{T}}_{e0}\left|n_{e}\rangle \right.\left|n_{e}\rangle \right.\left.\langle n_{e}\right|$ and $\sum_{n_{h}=1}^{n_{max}}\left.\langle n_{h}\right|{\hat{T}}_{h0}\left|n_{h}\rangle \right.\left|n_{h}\rangle \right.\left.\langle n_{h}\right|,$ respectively. $m_{ec}^{*}$ and $m_{hc}^{*}$ are the electron and hole effective masses in the QD core, respectively. The effective mass has different values between the QD core and shell. The effect of this difference on the kinetic energy is reflected in the perturbation terms ${\hat{T}}_{ep}$ and ${\hat{T}}_{hp}$ for the electron and hole, respectively. The perturbed electron kinetic energy operator ${\hat{T}}_{ep}$ is formulated as $-\frac{\hbar^{2}}{2m_{e\Delta }}\frac{\partial^{2}}{\partial x_{e}^{2}}$ in the shell region and zero in the core region, where $m_{e\Delta }=\frac{m_{ec}^{*}m_{es}^{*}}{m_{ec}^{*}+m_{es}^{*}}$ and $m_{es}^{*}$ is electron effective mass in the shell. The perturbed hole kinetic energy operator ${\hat{T}}_{hp}$ is formulated as $-\frac{\hbar^{2}}{2m_{h\Delta }}\frac{\partial^{2}}{\partial x_{e}^{2}}$ in the shell region and zero in the core region, where $m_{e\Delta }=\frac{m_{hc}^{*}m_{hs}^{*}}{m_{hc}^{*}+m_{hs}^{*}}$ and $m_{hs}^{*}$ is the hole effective mass in the shell. The perturbed kinetic energies for an electron and hole are represented by the momentum basis as $\sum_{n_{e1}=1}^{n_{max}}\sum_{n_{e2}=1}^{n_{max}}\left.\langle n_{e1}\right|{\hat{T}}_{ep}\left|n_{e2}\rangle \right.\left|n_{e1}\rangle \right.\left.\langle n_{e2}\right|$ and $\sum_{n_{h1}=1}^{n_{max}}\sum_{n_{h2}=1}^{n_{max}}\left.\langle n_{h1}\right|{\hat{T}}_{hp}\left|n_{h2}\rangle \right.\left|n_{h1}\rangle \right.\left.\langle n_{h2}\right|,$ respectively.

The momentum-basis matrix elements of total kinetic energy for an electron and hole are $T_{e,n_{e1}n_{e2}}=\left.\langle n_{e1}\right|{\hat{T}}_{e0}\left|n_{e2}\rangle \right.\delta_{n_{e1}n_{e2}}+\left.\langle n_{e1}\right|{\hat{T}}_{ep}\left|n_{e2}\right.\rangle$ and $T_{h,n_{h1}n_{h2}}=\left.\langle n_{h1}\right|{\hat{T}}_{h0}\left|n_{h2}\right.\rangle \delta_{n_{h1}n_{h2}}+\left.\langle n_{h1}\right|{\hat{T}}_{hp}\left|n_{h2}\right.\rangle$, respectively, where $\delta_{n_{e1}n_{e2}}$ and $\delta_{n_{h1}n_{h2}}$ are Kronecker delta functions. These momentum-basis matrix elements $T_{e,n_{e1}n_{e2}}$ and $T_{h,n_{h1}n_{h2}}$ can be transformed into position-basis matrix elements ${\tilde{T}}_{e,j_{e1}j_{e2}}$ and ${\tilde{T}}_{h,j_{h1}j_{h2}}$ through discrete Fourier transforms. Then, the position-basis matrix representation of the total kinetic energy, ${\hat{T}}_{e}+{\hat{T}}_{h}$, is obtained using the tensor products, as follows:

(7) $$\left(\sum_{j_{e1}=1}^{n_{max}}\sum_{j_{e2}=1}^{n_{max}}{\tilde{T}}_{e,j_{e1}j_{e2}}\left|j_{e1}\rangle \right.\left.\langle j_{e2}\right|\right)⨂{Ι}_{h}+{Ι}_{e}⨂\left(\sum_{j_{h1}=1}^{n_{max}}\sum_{j_{h2}=1}^{n_{max}}{\tilde{T}}_{h,j_{h1}{\tilde{j}}_{2}}\left|j_{h1}\rangle \right.\left.\langle j_{h2}\right|\right),$$

where ${Ι}_{e}$ and ${Ι}_{h}$ are the identity matrices. The matrix representation of ${\hat{V}}_{e}+{\hat{V}}_{h}$ is given as follows:

(8) $$\left(\sum_{j_{e}=1}^{n_{max}}\left.\langle j_{e}\right|{\hat{V}}_{e}\left|j_{e}\rangle \right.\left|j_{e}\rangle \right.\left.\langle j_{e}\right|\right)⨂{Ι}_{h}+{Ι}_{e}⨂\left(\sum_{j_{h}=1}^{n_{max}}\left.\langle j_{h}\right|{\hat{V}}_{h}\left|j_{h}\rangle \right.\left|j_{h}\rangle \right.\left.\langle j_{h}\right|\right).$$

In the position basis, the ${\hat{H}}_{int}$ matrix representation is as follows:

(9) $$\sum_{j_{e}=1}^{n_{max}}\sum_{j_{h}=1}^{n_{max}}\langle j_{e},\left.j_{h}\right|{\hat{H}}_{int}\left|j_{e},j_{h}\rangle \right.\left|j_{e},j_{h}\rangle \right.\langle j_{e},\left.j_{h}\right|$$

**Figure 1:**
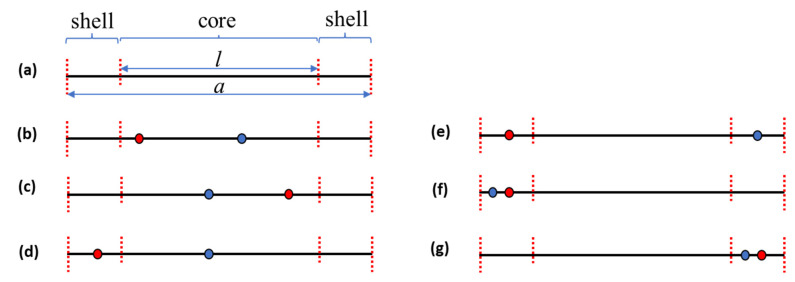
Diagrams showing the regions of a quantum dot (QD) (**a**). Positions of an electron and hole in a QD for case 1 (**b**), case 2 (**c**), case 3 (**d**), case 4 (**e**), case 5 (**f**), and case 6 (**g**). The filled circles in the figures represent an electron and hole.

Image charges [28] due to the heterogenous dielectric constant profile in a QD were considered when calculating Equation (9). The Coulomb energy of an electron–hole pair, $H_{int}$, was formulated for six cases, as follows:

(10) $$H_{int}=\{\begin{array}{ll} -\frac{e^{2}}{4\pi \epsilon_{c}}\left(\frac{1}{\left|x_{e}-x_{h}\right|}+\frac{\epsilon_{s}-\epsilon_{c}}{\epsilon_{s}+\epsilon_{c}}\frac{1}{\left|x_{e}+x_{h}-a+l\right|}\right), & \left(case\;1\right)\\ -\frac{e^{2}}{4\pi \epsilon_{c}}\left(\frac{1}{\left|x_{e}-x_{h}\right|}+\frac{\epsilon_{s}-\epsilon_{c}}{\epsilon_{s}+\epsilon_{c}}\frac{1}{\left|x_{e}+x_{h}-a-l\right|}\right), & \left(case\;2\right)\\ -\frac{\epsilon_{c}e^{2}}{2\pi \epsilon_{s}\left(\epsilon_{c}+\epsilon_{s}\right)}\frac{1}{\left|x_{e}-x_{h}\right|}, & \left(case\;3\right)\\ -\frac{\epsilon_{c}e^{2}}{\pi {\left(\epsilon_{c}+\epsilon_{s}\right)}^{2}}\frac{1}{\left|x_{e}-x_{h}\right|}, & \left(case\;4\right)\\ -\frac{e^{2}}{4\pi \epsilon_{s}}\left(\frac{1}{\left|x_{e}-x_{h}\right|}-\frac{\epsilon_{s}-\epsilon_{c}}{\epsilon_{s}+\epsilon_{c}}\frac{1}{\left|x_{e}+x_{h}-a+l\right|}\right), & \left(case\;5\right)\\ -\frac{e^{2}}{4\pi \epsilon_{s}}\left(\frac{1}{\left|x_{e}-x_{h}\right|}-\frac{\epsilon_{s}-\epsilon_{c}}{\epsilon_{s}+\epsilon_{c}}\frac{1}{\left|x_{e}+x_{h}-a-l\right|}\right), & \left(case\;6\right) \end{array},$$

where *a* and *l* represent the one-dimensional total length and core length of a QD, as shown in Figure 1a. $\epsilon_{c}$ and $\epsilon_{s}$ are QD core and shell dielectric constants, respectively. The individual cases of Equation (10) are described in the following: (case 1) both the electron and hole are in the QD core and closer to the left interface, as shown as Figure 1b; (case 2) both the electron and hole are in the QD core and closer to the right interface, as shown as Figure 1c; (case 3) the electron and hole are separated in both regions of the QD core and shell, as shown as Figure 1d; (case 4) the electron and hole are separated in each left and right shell region, as shown as Figure 1e; (case 5) both the electron and hole are in the left shell region, as shown as Figure 1f; and (case 6) both the electron and hole are in the right shell region, as shown as Figure 1g. The reciprocal of the electron–hole distance in the Coulomb energy equation exhibits a singularity when the distance is zero. The reciprocal of the distance, $1/\left|\Delta x\right|$, was modified to be $1/\left(\left|\Delta x\right|+d_{0}\right)$ to avoid the singularity. The correction factor $d_{0}$ was obtained by considering its relation to a three-dimensional electron–hole distance. The electron–hole distance in three dimensions is $\sqrt{\rho^{2}+{\left|\Delta x\right|}^{2}}$. ρ and $\left|\Delta x\right|$ represent the distance in the *y*-*z* plane and along the *x*-axis, respectively. Assuming that wavefunctions along the *x*-axis and in the *y*-*z* plane are independent and are spherically symmetric in the *y*-*z* plane, the *y*-*z* plane component of the reciprocal distance can be removed from the relation of $\frac{1}{d_{x}}=2\pi \int_{0}^{\infty }\frac{\rho {\left|\psi \left(\rho \right)\right|}^{2}}{\sqrt{\rho^{2}+{\left|\Delta x\right|}^{2}}}d\rho$. $1/d_{x}$ is the reciprocal *x*-axis distance averaged over the *y*-*z* plane and $\psi \left(\rho \right)$ is the wavefunction in the *y*-*z* plane. The approximated reciprocal distance, $1/\left(\left|\Delta x\right|+d_{0}\right)$, should approach $1/d_{x}$. For $\left|\Delta x\right|\to 0$, $1/\left(\left|\Delta x\right|+d_{0}\right)$ and $1/d_{x}$ approach $1/d_{0}$ and $1/a_{0}$, respectively, where $a_{0}=1/\left(2\pi \int_{0}^{\infty }{\left|\psi \left(\rho \right)\right|}^{2}d\rho \right)$. Hence, $d_{0}$ can be approximated as $a_{0}$. The Bohr model was used to approximate the $a_{0}$ value. For an electron–hole system, the energy operator of relative motion and Coulomb potential, ${\hat{E}}_{rel}$, is as follows:

(11) $${\hat{E}}_{rel}=-\frac{\hbar^{2}}{2\mu }\nabla_{rel}^{2}-\frac{e^{2}}{4\pi \epsilon r_{rel}},$$

where $\mu =\frac{m_{e}^{*}m_{h}^{*}}{m_{e}^{*}+m_{h}^{*}}$,$\nabla_{rel}^{2}$ is the Laplacian in relative coordinates, and $r_{rel}$ is the electron–hole distance. By applying the Bohr model for this system, the ground-state electron–hole distance is obtained as $r_{1}=\frac{4\pi \epsilon \hbar^{2}}{\mu e^{2}}$. $r_{1}$ is the value for three-dimensional motion, whereas $a_{0}$ corresponds to the two-dimensionally averaged electron–hole distance. Hence, $a_{0}$ was approximated as $a_{0}\approx \frac{\iint r_{1}sin\left(\theta \right)cos\left(\varphi \right)dA}{\iint dA}=\frac{4}{\pi^{2}}r_{1}$ where integration was conducted for the half sphere of radius $r_{1}$. θ and φ are polar and azimuthal angles, respectively. Thus, $d_{0}$ was approximated as $d_{0}\approx a_{0}\approx \frac{16\epsilon \hbar^{2}}{\pi \mu e^{2}}$. $m_{e}^{*}$, $m_{h}^{*}$, and ϵ of the QD core region were used for calculating the $d_{0}$ value.

The position basis representation of $\hat{H}$ was obtained by summing matrix representations of energy terms. The eigenvalues and eigenstates were obtained from the $\hat{H}$ by using Mathematica (Wolfram Research, Champaign, IL, USA) as a calculation tool. The total energies were obtained by summing up the *x*, *y*, and *z* one-dimensional energies.

## 3. Results and Discussion

**Figure 2:**
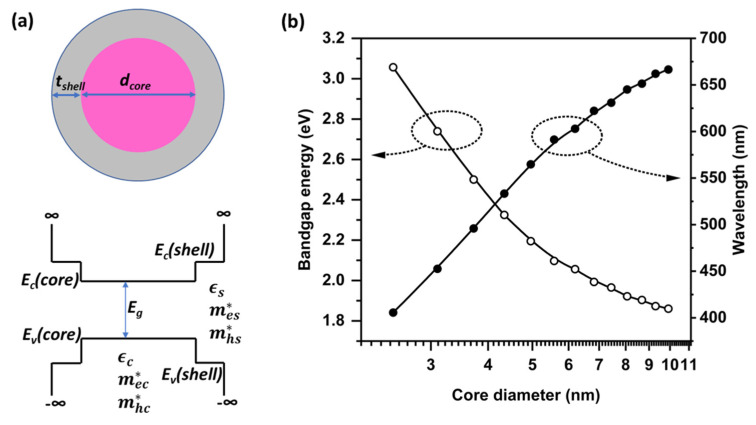
(**a**) Schematic diagram of a QD (top) and its potential profile (bottom), and (**b**) bandgap energy and wavelength as a function of the core diameter.

Quantum confinement effects were examined using the analysis method described above. The QDs with CdSe/ZnS core/shell structure were simulated. Figure 2a shows the QD structure and material parameters. The core diameter, *d_core_*, was estimated using the relation of $d_{core}/l=\sqrt[{3}]{6/\pi }$, where *l* is the one-dimensional core length; this relation was found by comparing the volumes of the spherical-shaped real QD and cube-shaped model QD used in this analysis. The QD core bandgap energy, *E_g_*, is 1.74 eV. At the interface between the core and shell, there are energy barriers of 1.27 eV for conduction band and 0.6 eV for valence band [29]. $m_{ec}^{*}$, $m_{hc}^{*}$, $m_{es}^{*}$, and $m_{es}^{*}$ are 0.13-, 0.45-, 0.34-, and 0.23-fold the electron rest mass, respectively [30]. The relative dielectric constant is 6.36 in the core and 5.71 in the shell [31,32]. The shell thickness, *t_shell_*, was fixed at 2.5 nm [23]. The bandgap energy and emission light wavelength as a function of QD diameter are shown in Figure 2b. As the core diameter increased, the bandgap energy decreased, and the wavelength increased. These behaviors are explained by quantum confinement effects. The core diameter of a CdSe/ZnS QD emitting a 530 nm green light was 4.2 nm; thus, the total diameter of the QDs was 9.2 nm, which accords with previous experimental reports [33,34].

**Figure 3:**
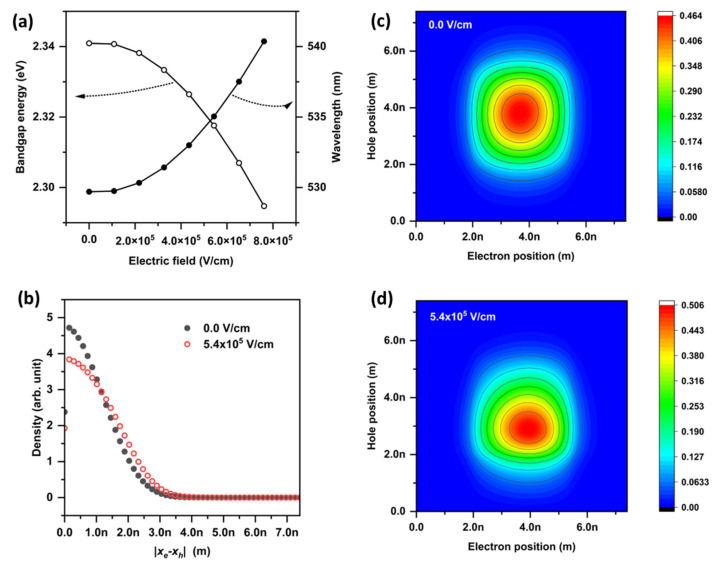
(**a**) Bandgap energy and wavelength as a function of the external electric-field intensity, (**b**) probability densities as a function of the one-dimensional electron–hole distance, and ground state wavefunction distributions as a function of electron and hole positions (**c**) without and (**d**) with external electric field. The legend shows the external electric-field intensity values.

The dependency of bandgap energy and wavelength on the external electric field intensity are shown in Figure 3a. The core diameter and shell thickness were 4.2 and 2.5 nm, respectively, so that the QD diameter was 9.2 nm. The external electric field was applied along the negative direction. As the external electric field intensity increased, the bandgap energy decreased, and the light emission wavelength increased. This behavior accords with previous experimental results and is explained by the QCSE. A 4.7 × 10^7^ V/cm electric field resulted in a 4 nm redshift in the light emission wavelength. The redshift of about 4 nm was easily observed from QLEDs with CdSe/ZnS QD EMLs [34,35]. The electric field of 4.7 × 10^7^ V/cm corresponds to a 0.43 V voltage drop across the 9.2 nm diameter QD. Thus, for a well-fabricated QLED, most of the bias voltage is applied to the charge transport layers and only a small fraction of the bias voltage is applied to the QD. The one-dimensional probability densities of ground states as a function of electron–hole distance, with and without external electric field, are shown in Figure 3b. The expected value of the electron–hole distance increased with electric field application, as shown in the figure. More detailed behaviors of carriers in ground states are shown in Figure 3c,d. The electron and hole positions were almost symmetric without an electric field (Figure 3c); however, the symmetricity was broken with an electric-field application (Figure 3d).

**Figure 4:**
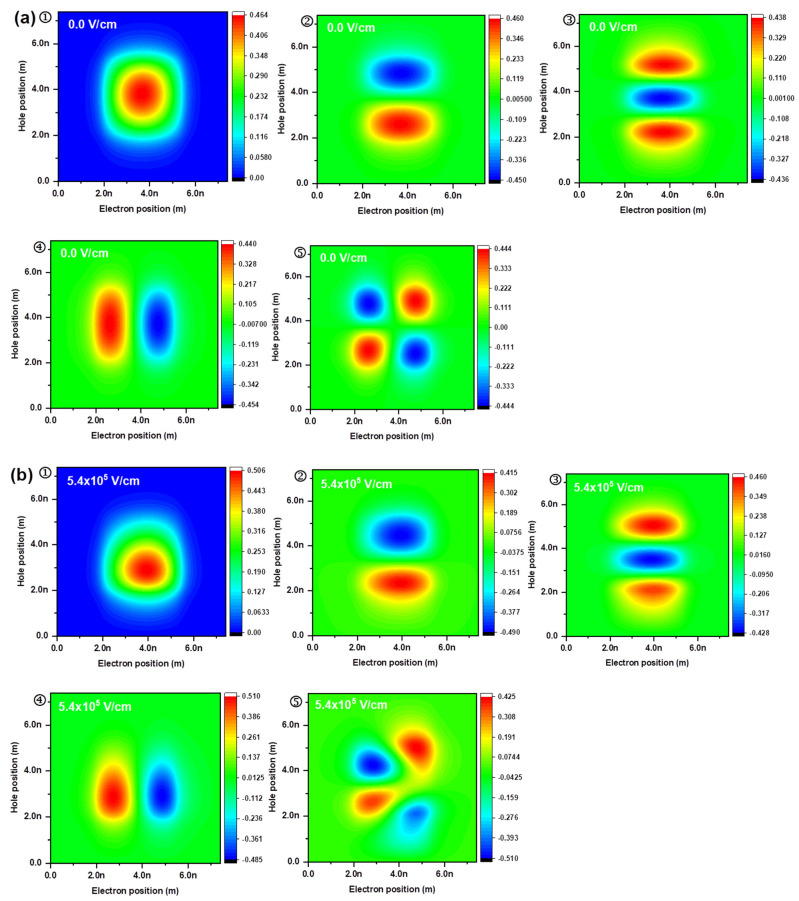
Spatial wavefunction distributions (**a**) without and (**b**) with an external electric field of $5.4\times 10^{5}\;V/\mathrm{cm}$. In the figures, ①–⑤ indicate the first, second, third, fourth, and fifth lowest energy state, respectively.

Wavefunction distributions as a function of electron and hole positions are shown in Figure 4; the wavefunctions with the lowest five energy levels are shown in the figure. Without an external electric field (Figure 4a), the first and fifth states showed symmetric distributions. For the second and third states, the electron distribution was concentrated at the center region, and the hole distribution was dispersed along the axis; for the fourth state, the hole distribution was concentrated in the center region, and the electron distribution was dispersed along the axis. The smaller effective mass of the electron allowed it to concentrate around the center, whereas the heavier hole was dispersed along the axis in the lower energy states (second and third states). With an external electric field along the negative direction (Figure 4b), the electron and hole moved in positive and negative directions, respectively, compared with those without an external electric field.

## 4. Conclusions

A QD energy level simulation was designed to reproduce a quantum mechanical analytic solution based on perturbation theory. The simulation was conducted using a numerical method. Using this approach, the electron and hole behavior in a QD could be analyzed effectively based on the Schrödinger equation, with appropriate consideration of the core/shell structure and material properties. As this approach imitates the analytic perturbation method, the results are equivalent to analytic solutions based on a perturbation method. Owing to the effectiveness of the proposed method, QD behavior according to the core diameter and external electric-field intensity could be analyzed reliably and easily. The light-emission wavelength dependency on the core diameter was examined; a 9.2 nm diameter CdSe/ZnS QD with a 4.2 nm diameter core and a 2.5 nm thick shell emitted a 530 nm green light. The electric-field-induced redshift was examined and resulted in a ~4 nm shift at $5.4\times 10^{5}$ V/cm electric-field intensity. The QD diameter, amount of redshift, and electric-field intensity for the redshift agree well with previously reported experimental results. In addition to the energy levels and light emission wavelengths, the spatial distributions of wavefunctions were resolved; this information helps explain carrier behavior in a QD, which could lead to improved QD and QLED structures. Thus, this analysis method is widely applicable for studying QD characteristics for various structures and material compositions. Despite the success of this work, it is desirable to conduct additional studies to improve the analysis method further, such as by calculating transition probabilities between the quantum-confined states.

## Data Availability

Not applicable.
